# Prevalence, sleep characteristics, and comorbidities in a population at high risk for obstructive sleep apnea: A nationwide questionnaire study in South Korea

**DOI:** 10.1371/journal.pone.0193549

**Published:** 2018-02-28

**Authors:** Jun-Sang Sunwoo, Young Hwangbo, Won-Joo Kim, Min Kyung Chu, Chang-Ho Yun, Kwang Ik Yang

**Affiliations:** 1 Department of Neurology, Soonchunhyang University College of Medicine, Seoul Hospital, Seoul, South Korea; 2 Department of Preventive Medicine, Soonchunhyang University College of Medicine, Cheonan, South Korea; 3 Department of Neurology, Gangnam Severance Hospital, Yonsei University, College of Medicine, Seoul, South Korea; 4 Department of Neurology, Hallym University College of Medicine, Seoul, South Korea; 5 Department of Neurology, Bundang Clinical Neuroscience Center, Seoul National University Bundang Hospital, Seongnam, South Korea; 6 Sleep Disorders Center, Department of Neurology, Soonchunhyang University College of Medicine, Cheonan Hospital, Cheonan, South Korea; University of Rome Tor Vergata, ITALY

## Abstract

**Objective:**

To determine the prevalence, sleep characteristics, and comorbidities associated with a high risk for obstructive sleep apnea (OSA) in the Korean adult population.

**Methods:**

We analyzed data from 2,740 subjects who responded to a nationwide questionnaire survey of sleep characteristics. Those who qualified under two or more symptom categories of the Berlin questionnaire were defined as “at high risk for OSA”. We investigated their socio-demographic information, sleep habits, and medical and psychiatric comorbidities. Logistic regression analyses were performed to identify factors and consequences significantly associated with a high risk for OSA.

**Results:**

The prevalence of a high risk for OSA was 15.8% (95% confidence interval [CI] 14.5–17.2%). Multiple logistic regression analysis showed that old age (≥ 70 years, odds ratio [OR] 2.68) and body mass index ≥ 25 kg/m^2^ (OR 10.75) were significantly related with a high risk for OSA, whereas regular physical activity (OR 0.70) had a protective effect. Subjective sleep characteristics associated with a high risk for OSA were perceived insufficient sleep (OR 1.49), excessive daytime sleepiness (OR 1.88), and insomnia (OR 3.70). In addition, hypertension (OR 5.83), diabetes mellitus (OR 2.54), hyperlipidemia (OR 2.85), and anxiety (OR 1.63) were comorbid conditions independently associated with a high risk for OSA.

**Conclusions:**

This is the first study to demonstrate the prevalence of a high risk for OSA in a nationwide representative sample of the Korean adult population. These findings elucidate the epidemiology and clinical characteristics of those at high risk for OSA.

## Introduction

Obstructive sleep apnea (OSA) is a common sleep disorder characterized by repetitive upper airway collapse during sleep with consequent oxygen desaturation, frequent arousals, and sleep fragmentation [1]. Of particular importance is that untreated OSA significantly increases the risk of cardiovascular diseases, stroke, and death [2, 3]. In addition, OSA leads to neurocognitive consequences including excessive daytime sleepiness, reduced cognitive performance, and increased risk for motor vehicle and work accidents [4, 5]. To prevent the health consequences of OSA, early identification and optimal treatment of OSA is necessary. The prevalence of OSA varies with measurement methods, diagnostic criteria, and apnea-hypopnea index (AHI) cutpoints [6]. Previous cohort studies with in-laboratory polysomnography (PSG) demonstrated that the prevalence of OSA defined by AHI ≥ 5 ranged from 17 to 26% in men and from 9 to 28% in women [7–10]. Similarly, 27% of men and 17% of women in the Korean adult population were found to have an AHI of 5 or more [11]. Furthermore, OSA is more prevalent in patients with resistant hypertension and cardiovascular diseases, but OSA remains unrecognized and untreated in most patients [12, 13].

PSG is considered the gold standard for diagnosis of OSA in adults [14]. However, considering the high prevalence of OSA, PSG testing of all patients suspected of having OSA is not feasible due to significant cost and limited accessibility. OSA needs to be screened for in any patients with OSA symptoms, such as witnessed apnea, snoring, nocturnal gasping, and unexplained daytime sleepiness, and those who have comorbid conditions related to a high risk of OSA, such as obesity, heart failure, hypertension, and stroke [15]. Then, those found to be at high risk should undergo objective sleep testing to confirm the diagnosis as well as to determine the severity of OSA. However, OSA symptoms are not adequately screened or assessed in primary care settings [16]. Clinical questionnaires can be a convenient and efficient means of screening individuals at high risk of OSA. The Berlin questionnaire is the most widely used questionnaire for screening for a high risk of OSA in clinical practice [17–19], and its screening properties have been validated in several population-based studies [20–22]. The diagnostic performance of the Berlin questionnaire was shown to have a pooled sensitivity of 0.76 and a pooled specificity of 0.45 when predicting OSA with an AHI cutoff of ≥ 5 [14].

In the present study, we determined the prevalence of a high risk for OSA estimated by the Berlin questionnaire in a nationwide sample representative of the Korean adult population. In addition to the risk for OSA, we collected data about subjective sleep characteristics and comorbid medical conditions from the study subjects. Based on this data, we determined the factors and health consequences independently and significantly associated with a high risk for OSA.

## Methods

### Subjects

A nationwide questionnaire survey for subjective sleep characteristics was performed for adults aged ≥ 19 years. Study population sampling and the questionnaire survey were conducted by Gallup Korea and the detailed procedures have been described elsewhere [23, 24]. Briefly, Gallup Korea approached a total of 7,615 adults in 2010. The sampling areas included all 15 administrative districts except for Jeju province: 8 provinces, 6 metropolitan cities, and the Seoul special city. Consequently, 2,836 (37.2%) subjects responded to the questionnaire through face-to-face interviews. Among them, we excluded 96 subjects who reported incomplete data for sleep habits (n = 42) and socio-demographic information (n = 54). All participants provided written informed consent before responding to the survey. Data collected from the questionnaire survey were de-identified to protect the privacy of study subjects. The study protocol was approved by the Institutional Review Board of Soonchunhyang University Cheonan Hospital (IRB No. 2017-03-028) and was conducted according to the Declaration of Helsinki and the Good Clinical Practice guidelines.

### Risk stratification for obstructive sleep apnea

We estimated the risk of OSA of the study population by using the Berlin questionnaire [25]. The Korean version of the Berlin questionnaire was previously developed and its usefulness as a screening tool for OSA was validated in an adult population [26]. The Berlin questionnaire consists of three symptom categories. Briefly, category 1 evaluates snoring and sleep apnea, while category 2 addresses daytime sleepiness and fatigue. Category 3 investigates the presence of hypertension or obesity defined as body mass index (BMI) ≥ 25 kg/m^2^ according to the scoring guideline of the Korean version of the Berlin questionnaire [26, 27]. Subjects who qualify for two or more symptom categories were classified as at high risk for OSA. Conversely, those who report positive symptom categories of ≤ 1 were classified as “at low risk for OSA”.

### Subjective sleep characteristics

Subjects were asked to report sleep habits over the last month, such as wake-up time, bedtime, sleep latency, and night sleep duration separately for weekdays and weekends. Average sleep duration was calculated as follows: (sleep duration on weekdays × 5 + sleep duration on weekends × 2)/7. When subjects slept longer on weekends than on weekdays, we measured weekend catch-up sleep by subtracting sleep duration on weekdays from sleep duration on weekends. Chronotype was determined by measuring the mid-sleep time on free days corrected for oversleep on free days (MSFsc), which was calculated based on the methods used in a previous study [28]. We also investigated perceived insufficient sleep, unmet sleep need, the Epworth sleepiness scale (ESS), the Pittsburgh sleep quality index (PSQI), and the insomnia severity index (ISI) as previously described [29].

### Other investigations

We investigated socio-demographic information, such as age, sex, BMI, education level, occupation, income level, alcohol consumption, smoking status, and physical activity. The detailed protocols have been described elsewhere [29]. In addition, we evaluated past medical history of hypertension, diabetes mellitus, hyperlipidemia, myocardial infarction, angina pectoris, other heart diseases, and stroke. Among them, stroke, myocardial infarction, angina pectoris, and other heart diseases were combined into the category of cardiovascular diseases. Furthermore, as screening tools for psychiatric comorbidity, we used the Goldberg anxiety scale (GAS) and the Patient Health Questionnaire-9 (PHQ-9), respectively [30, 31].

### Statistical analysis

Comparisons of continuous variables between high- and low-risk groups for OSA were conducted by the Student’s t-test, while comparisons of categorical variables were performed by the Pearson’s chi-square test. Unadjusted odds ratio (OR) and 95% confidence interval (CI) were estimated by univariable logistic regression analysis for each predictor variable. The dependent variable was set as high risk for OSA estimated by the Berlin questionnaire. Next, we performed multiple logistic regression analysis to identify independent associations between predictor variables and high risk for OSA. Predictor variables with P < 0.05 from the univariable logistic regression analysis and potential confounders were included as covariates for adjustment. In addition, a linear trend in the adjusted ORs of the predictor variables was estimated by the likelihood ratio test. A two-tailed P < 0.05 was considered statistically significant. All statistical analyses were performed with SPSS version 18 (SPSS Inc., Chicago, IL).

## Results

### Prevalence of high risk for OSA

**Fig 1:**
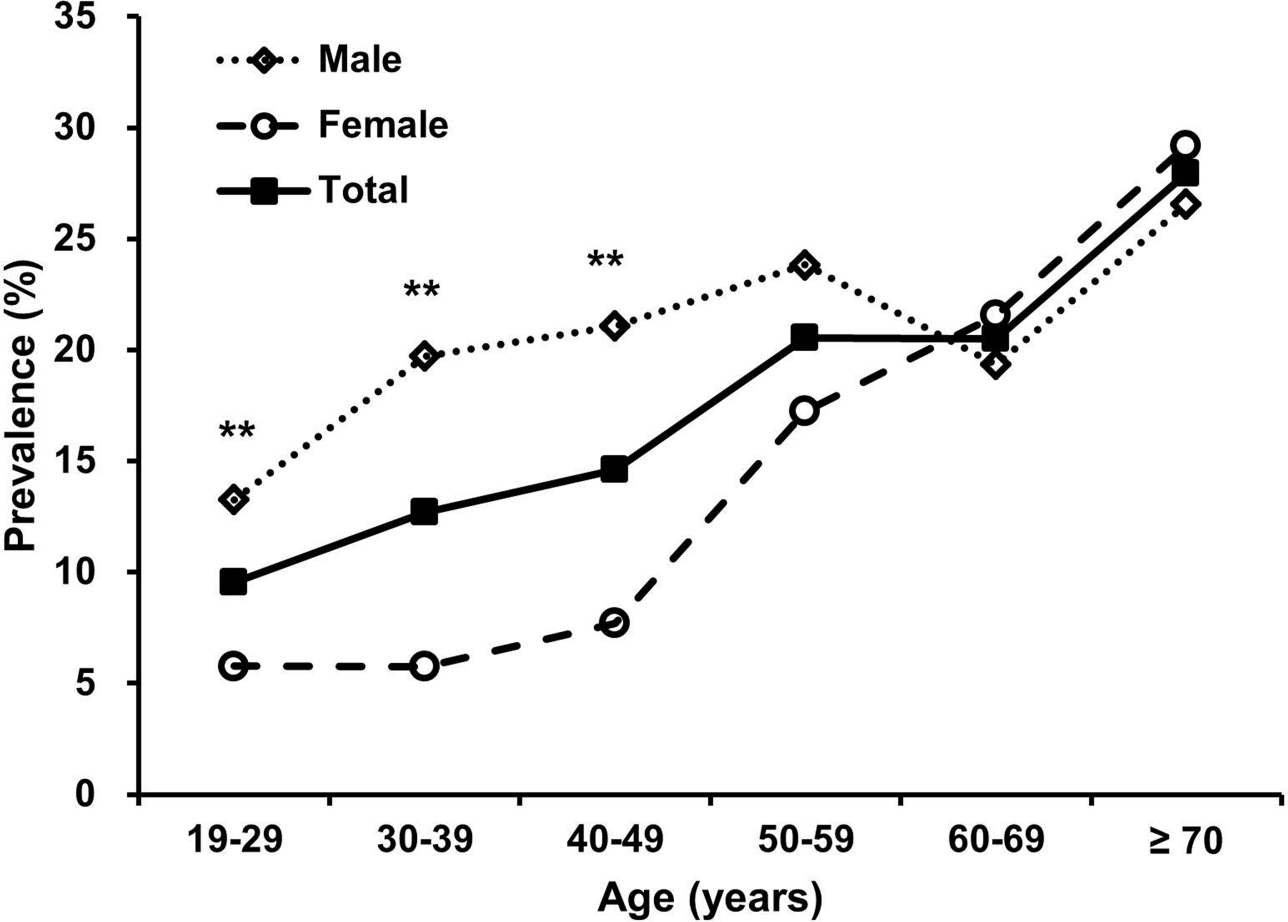
Prevalence of a high risk for obstructive sleep apnea according to age and sex. High risk for obstructive sleep apnea was defined as positive symptom categories of ≥ 2 on the Berlin questionnaire. *P < 0.05 and **P < 0.01 for comparisons between male and female in each age group. n = 1,368 in male and n = 1,372 in female.

Data collected from a total of 2,740 subjects were analyzed in this study. Their mean age was 44.5 ± 15.0 years and 49.9% were men. Based on the risk stratification by the Berlin questionnaire, the overall prevalence of a high risk for OSA was 15.8% (434 of 2,740; 95% CI 14.5–17.2%). The prevalence of high-risk group of OSA in men (19.8%, 95% CI 17.7–21.9%) was higher than that in women (11.9%, 95% CI 10.4–13.7%; P < 0.001). As shown in Fig 1, the prevalence increased with age (linear by linear association, P < 0.001). When subjects were further stratified by age, those 19–29, 30–39, and 40–49 years of age showed a higher prevalence in men than in women (P = 0.004, < 0.001, and < 0.001, respectively). However, a high risk for OSA was equally distributed between men and women in those aged 60 years or more.

### Comparisons between high- and low-risk groups

The distribution of the study population and the prevalence of a high risk for OSA according to socio-demographic variables are summarized in Table 1. Univariable analysis demonstrated that subjects at high risk for OSA were more likely to be older, male, obese, less educated, and low-income. Safety accidents at work were also associated with a high risk for OSA (unadjusted OR 1.78, P = 0.021). However, shift work and physical activity did not significantly influence the risk for OSA. Compared to never smokers, both ex-smokers (unadjusted OR 1.94, P < 0.001) and current smokers (unadjusted OR 1.67, P < 0.001) showed a higher proportion of a high risk for OSA. Those who drank alcohol ≥ 2 days per week also had an increased odds of a high risk for OSA (unadjusted OR 1.38, P = 0.016) compared to never drinkers.

**Table 1:** Prevalence of high risk for obstructive sleep apnea according to socio-demographic characteristics and comorbidity (n = 2,740).

Variables	Categories	Total No.	High risk of OSA	Unadjusted OR (95% CI)
			No. (%)	
Age, yr	< 30	524	50 (9.5)	1.00
	30–39	590	75 (12.7)	1.38 (0.95–2.02)
	40–49	589	86 (14.6)	1.62 (1.12–2.35)
	50–59	511	105 (20.5)	2.45 (1.71–3.52)
	60–69	390	80 (20.5)	2.45 (1.67–3.58)
	≥ 70	136	38 (27.9)	3.68 (2.29–5.91)
Sex	Female	1372	163 (11.9)	1.00
	Male	1368	271 (19.8)	1.83 (1.48–2.26)
BMI, kg/m^2^	< 18.5	126	2 (1.6)	0.21 (0.05–0.85)
	18.5–25	1969	141 (7.2)	1.00
	≥ 25	645	291 (45.1)	10.66 (8.46–13.43)
Education	≤ Middle school	494	117 (23.7)	1.60 (1.24–2.07)
	High school	1200	195 (16.3)	1.00
	≥ College	1046	122 (11.7)	0.68 (0.53–0.87)
Occupation	Unemployed	1008	139 (13.8)	1.00
	Self-employment	432	96 (22.2)	1.79 (1.34–2.38)
	Sales and service	471	67 (14.2)	1.04 (0.76–1.42)
	Manual labor	316	60 (19.0)	1.47 (1.05–2.04)
	Office work	513	72 (14.0)	1.02 (0.75–1.39)
Shift work	No	2168	340 (15.7)	1.00
	Yes	145	25 (17.2)	1.12 (0.72–1.75)
Accident at work	No	2651	412 (15.5)	1.00
	Yes	89	22 (24.7)	1.78 (1.09–2.92)
Income level	Low	786	167 (21.2)	1.68 (1.33–2.14)
	Middle	1181	163 (13.8)	1.00
	High	689	91 (13.2)	0.95 (0.72–1.25)
Alcohol drinking	None	955	147 (15.4)	1.00
	≤ 1/week	1153	160 (13.9)	0.89 (0.70–1.13)
	≥ 2/week	632	127 (20.1)	1.38 (1.06–1.80)
Smoking	Never	1661	213 (12.8)	1.00
	Ex-smoker	343	76 (22.2)	1.94 (1.44–2.59)
	Current	736	145 (19.7)	1.67 (1.32–2.10)
Physical activity	None	1444	242 (16.8)	1.00
	1–2/week	563	85 (15.1)	0.88 (0.68–1.16)
	≥ 3/week	733	107 (14.6)	0.85 (0.66–1.09)
Hypertension	No	2387	283 (11.9)	1.00
	Yes	353	151 (42.8)	5.56 (4.35–7.10)
Diabetes mellitus	No	2605	382 (14.7)	1.00
	Yes	135	52 (38.5)	3.65 (2.54–5.24)
Hyperlipidemia	No	2660	404 (15.2)	1.00
	Yes	80	30 (37.5)	3.35 (2.10–5.33)
Cardiovascular diseases*	No	2653	408 (15.4)	1.00
	Yes	87	26 (29.9)	2.35 (1.46–3.76)
Depression^†^	No	2611	391 (15.0)	1.00
	Yes	129	43 (33.3)	2.84 (1.94–4.16)
Anxiety^†^	No	2422	342 (14.1)	1.00
	Yes	318	92 (28.9)	2.48 (1.89–3.24)

Data for shift work and income level were available in 2,313 and 2,656 subjects, respectively. Unadjusted odds ratio was calculated by univariable logistic regression analysis for each predictor variable. Abbreviations: BMI, body mass index; OR, odds ratio.

*Cardiovascular diseases include myocardial infarction, stroke, angina, and other heart disease.

^†^Depression was defined as the Patient Health Questionnaire-9 score of ≥ 10, and anxiety was defined as the Goldberg Anxiety Scale score of ≥ 5.

We compared subjective sleep characteristics between high- and low-risk groups for OSA (Table 2). Average sleep duration of the high-risk group (7.0 ± 1.4 h) was shorter than that of the low-risk group (7.4 ± 1.2 h, P < 0.001). Although weekend catch-up sleep of ≥ 1 h was less frequently observed in the high-risk group than in the low-risk group (29.5 vs. 38.2%, P = 0.001), there was no significant difference in the duration of weekend catch-up sleep (1.8 ± 1.1 vs. 1.8 ± 1.1 h, P = 0.963). Furthermore, sleep characteristics associated with a high risk for OSA included porlonged sleep latency, higher prevalence of perceived insufficient sleep, excessive daytime sleepiness, poor sleep quality, and insomnia (P < 0.001 for all).

**Table 2:** Comparison of subjective sleep characteristics between high- and low-risk groups for obstructive sleep apnea.

Variables	High risk (n = 434)	Low risk (n = 2306)	P
Sleep duration, h			
Average	7.0 ± 1.4	7.4 ± 1.2	< 0.001
Weekday	6.8 ± 1.5	7.2 ± 1.2	< 0.001
Weekend	7.3 ± 1.7	7.8 ± 1.5	< 0.001
Weekend catch-up sleep ≥ 1 h	128 (29.5)	881 (38.2)	0.001
Sleep latency, min	27.9 ± 27.4	23.6 ± 22.4	0.002
MSFsc, h*	3.8 ± 1.8	3.9 ± 1.5	0.201
Perceived insufficient sleep	175 (40.3)	666 (28.9)	< 0.001
Excessive daytime sleepiness	97 (22.4)	228 (9.9)	< 0.001
Poor sleep quality	155 (35.7)	419 (18.2)	< 0.001
Insomnia severity index (ISI)			< 0.001
Normal	306 (70.5)	1961 (85.0)	
Subthreshold insomnia	79 (18.2)	266 (11.5)	
Clinical insomnia	49 (11.3)	79 (3.4)	

Data are presented as mean ± standard deviation or number (%). Excessive daytime sleepiness was defined as the Epworth sleepiness scale score of > 10, and poor sleep quality was defined as the Pittsburgh sleep quality index score of > 5. We defined insomnia as follows: subthreshold (ISI score 8–14) and clinical insomnia (ISI score ≥ 15). Abbreviations: MSFsc, mid-sleep time on free days corrected for oversleep on free days (local time in hours after midnight).

*Chronotype data were available in 2,736 subjects.

### Multivariable analysis for a high risk of OSA

We performed multiple logistic regression analysis to determine the factors independently associated with a high risk for OSA. In this model, predictor variables included age, sex, BMI, occupation, education and income level, alcohol consumption, and smoking status. Shift work and physical activity were also included as covariates. Consequently, we identified that old age (≥ 70 years, OR 2.68), BMI ≥ 25 kg/m^2^ (OR 10.75), and regular physical activity (OR 0.70) were significantly and independently associated with a high risk for OSA (Table 3). In addition, there was a trend towards an increased risk for OSA in people on low incomes (OR 1.39, 95% CI 0.99–1.94, P = 0.056). However, there was no significant association with other factors including sex, education, occupation, smoking status, and alcohol consumption. There was no significant multicollinearity among predictor variables with the variance inflation factors ranging from 1.06 to 2.25.

**Table 3:** Risk factors associated with high risk of obstructive sleep apnea.

Variables	Adjusted OR (95% CI)
Age, yr (vs. 19–29)	
30–39	0.97 (0.60–1.55)
40–49	1.08 (0.67–1.75)
50–59	1.50 (0.90–2.49)
60–69	1.13 (0.62–2.06)
≥ 70	2.68 (1.24–5.82)*
BMI, kg/m^2^ (vs. 18.5–25)	
< 18.5	0.30 (0.07–1.25)
≥ 25	10.75 (8.21–14.06)**
Physical activity (vs. none)	
1–2/week	0.72 (0.51–1.01)
≥ 3/week	0.70 (0.51–0.97)*

Adjusted odds ratios were calculated by multivariable logistic regression analysis. Covariates included sex, education, occupation, income level, shift work, alcohol consumption, and smoking status. Abbreviations: OR, odds ratio; CI, confidence interval; BMI, body mass index.

*P < 0.05

**P < 0.01.

Next, we constructed a multiple logistic regression model to evaluate consequences associated with a high risk for OSA. Predictor variables included subjective sleep characteristics showing significant differences between the two groups, safety accidents at work, and medical conditions such as hypertension, diabetes, hyperlipidemia, cardiovascular diseases, depression, and anxiety. In addition, we entered socio-demographic variables to control for confounding. As shown in Table 4, perceived insufficient sleep (OR 1.49), excessive daytime sleepiness (OR 1.88), and insomnia (subthreshold, OR 1.95; clinical OR 3.70; P for linear trend < 0.001) remained significantly associated with a high risk for OSA. Poor sleep quality (OR 1.51, 95% CI 0.97–2.36) was likely to increase the odds of being at high risk for OSA, but it failed to reach a significance level (P = 0.071). Moreover, the presence of hypertension (OR 5.83), diabetes mellitus (OR 2.54), hyperlipidemia (OR 2.85), and anxiety (OR 1.63) had independent associations with a high risk for OSA. However, the associations with cardiovascular diseases, safety accidents, and depression were not significant. The variance inflation factors of all of the predictor variables included in this model ranged between 1.06 and 2.45, suggesting that there were no significant problems with multicollinearity.

**Table 4:** Sleep characteristics and comorbidity associated with high risk of obstructive sleep apnea.

Variables	Adjusted OR (95% CI)
Perceived insufficient sleep	1.49 (1.06–2.10)*
Excessive daytime sleepiness	1.88 (1.27–2.77)**
Insomnia	
Subthreshold	1.95 (1.23–3.10)**
Clinical	3.70 (1.75–7.85)**
Hypertension	5.83 (3.91–8.69)**
Diabetes mellitus	2.54 (1.46–4.42)**
Hyperlipidemia	2.85 (1.36–5.95)**
Anxiety	1.63 (1.03–2.59) *

The multivariable logistic regression model was adjusted for age, sex, body mass index, education, occupation, shift work, safety accidents, income level, alcohol consumption, smoking status, physical activity, average sleep duration, sleep latency, weekend catch-up sleep (≥ 1 h), poor sleep quality, depression, and cardiovascular diseases. Abbreviations: OR, odds ratio; CI, confidence interval.

*P < 0.05

**P < 0.01.

## Discussion

Our data collected from a nationwide, population-based survey demonstrated that 15.8% of adults were at high risk of OSA based on the Berlin questionnaire. Previous data showed that the prevalence of high risk group of OSA was 12.4% in Korean adults [27], which is slightly lower than that found in our study. However, that prior study only targeted South Gyeongsang province, which is one of the 8 provinces in Korea. Accordingly, our data is the first to demonstrate the prevalence of a high risk for OSA in a nationwide representative sample of the Korean adult population. Furthermore, we thoroughly investigated the association of a high risk for OSA with various sleep characteristics and comorbidities, which is another strength of this study.

Several studies using the Berlin questionnaire have been conducted in other countries. Data from the Norwegian and the United States populations showed prevalence of a high risk for OSA of 24.3% and 26%, respectively [20, 21], which is higher than that reported in the present study. Because excess body weight is the strongest risk factor for OSA [32], the differences in prevalence of obesity among the study populations might account for the discrepancies in the prevalence results. Consistent with this, BMI > 30 kg/m^2^ was noted in 25% and 14.8% of the screening samples in the United States and Norwegian studies, respectively, whereas only 2.1% (57 of 2,740) were identified in our study. Another possible explanation is a different age distribution among the study populations, considering that old age is a significant risk factor of OSA [33]. In this regard, the mean age of the screening samples was 44.5 years in this study, which is younger than the 47.8 and 49 years in previous studies. However, the prevalence of PSG-confirmed OSA in Koreans was reported to be 4.5% in men and 3.2% in women when OSA was defined as an AHI ≥ 5 plus excessive daytime sleepiness [11], which is comparable to that found in Caucasians [7, 8]. Therefore, any discrepancies in the questionnaire-based prevalence among the study population might be attributed to correlates of OSA rather than the disease itself. Although the Berlin questionnaire is useful for screening subjects at high risk of OSA [26], it should be kept in mind that the questionnaire survey cannot be interchangeable with PSG for the diagnosis of OSA.

In this study, old age and BMI ≥ 25 kg/m^2^ were independent factors associated with a high risk OSA. This is in close agreement with previous observations that the prevalence of OSA increases with age and excess body weight [8, 9, 34]. Notably, we found that at least three times a week of regular physical activity significantly reduced the risk for OSA after adjusting for BMI and other confounding covariates. Previous epidemiologic studies also demonstrated the protective association of regular physical activity against sleep-disordered breathing [35, 36]. Consistent with our finding, the protective effect of regular physical activity on OSA was reported to be independent of body habitus [37]. Furthermore, a recent meta-analysis showed that exercise training significantly improved sleep efficiency, cardiovascular fitness, and daytime sleepiness as well as AHI although there was no significant reduction in BMI [38]. Given the major contribution of comorbid hypertension to the high risk of OSA on the Berlin questionnaire, it is also possible that the beneficial effect of regular exercise was mediated by its blood pressure lowering effect [39–41].

A higher prevalence of OSA in men compared with women has been established from previous epidemiologic studies [6, 8]. In agreement with this, the unadjusted prevalence of high risk for OSA in men was 1.83-fold higher than that in women in this study. However, male sex was not found to be an independent factor for predicting high risk of OSA in the multivariable analysis. As shown in Fig 1, the significant male predominance in high risk for OSA disappeared after age 50 years. Previous population-based studies demonstrated similar results that sex differences in the prevalence of OSA in people older than 65 years were relatively small compared with those in middle age [7, 42, 43]. This phenomenon might be partially accounted for by the increase in the OSA risk in postmenopausal women [7, 44]. It is also possible that the higher mortality rate associated with OSA causes death in men more often than in women [45, 46], which relatively decreases the prevalence of OSA in men in older populations.

We found that hypertension, diabetes mellitus, and hyperlipidemia were comorbid conditions independently associated with a high risk for OSA. It has been well-established that OSA is implicated in cardiovascular diseases and notably hypertension [3, 47]. Longitudinal data from the Wisconsin Sleep Cohort Study indicated that moderate or severe OSA had a 3-fold increased risk for the presence of hypertension at the 4-year follow-up [48]. Moreover, CPAP treatment for 12 weeks significantly decreased 24 h mean blood pressure compared to the control [49]. In agreement with our observations, accumulating evidence has supported the association of OSA with diabetes mellitus and insulin resistance [50–53]. Although clinical evidence that OSA is associated with hyperlipidemia is relatively sparse [54, 55], experimental data suggested that intermittent hypoxia induces hyperlipidemia and atherosclerosis [56, 57]. In terms of psychiatric comorbidity, a high risk of OSA was significantly associated with anxiety. Our observation supports previous studies showing that patients with sleep disordered breathing had a higher prevalence of anxiety than controls [58, 59]. Beneficial effects of positive airway pressure (PAP) therapy on quality of life and anxiety in OSA patients also substantiate the interaction between anxiety and OSA [60, 61].

Perceived insufficient sleep is a sleep characteristic not only affected by quantitative sleep deprivation but also reflecting the presence of underlying sleep disorders such as OSA [62]. Consistent with this, the association between perceived insufficient sleep and a high risk for OSA was significant in our data, independent of average sleep duration. Considering sleep fragmentation with repeated arousals in OSA [63], perceived insufficient sleep and excessive daytime sleepiness would be inevitable consequences of OSA. In addition, it is noteworthy that insomnia was independently associated with a high risk for OSA in this study. The dose-response relationship between insomnia severity and ORs for high risk of OSA confirmed the interaction between the two conditions. There has been accumulating evidence to support comorbid insomnia in patients with OSA [64, 65]. Previous studies reported that insomnia coexists in 39%–55% of patients with OSA [66]. Although mechanisms of the comorbid relationship between the two sleep disorders are not fully understood, it is presumed that frequent arousals with increased sympathetic and hypothalamic-pituitary-adrenal axis activity resulting from OSA may precipitate or exacerbate insomnia symptoms [67].

It has been well established that OSA is significantly associated with an increased risk of occupational accidents, particularly motor vehicle accidents [68, 69]. A recent meta-analysis showed that workers with suspected OSA have an approximately twofold increased odds of work-related accidents compared to those without OSA [5]. However, the association between a high risk for OSA and safety accidents at work was not found to be significant in our multivariate analysis. A possible explanation for this discrepancy is that our study did not include a sufficient number of professional drivers. The effect size for non-driving accidents was significantly smaller than that obtained for driving accidents [5]. Moreover, our study investigated a variety of potential comorbidities of a high risk for OSA rather than focusing on the risk for occupational accidents. Therefore, other covariates included in the multivariate analysis might have contributed to the different result for risk of safety accidents. Our findings should not be mistakenly interpreted that there is no possible association between a high risk for OSA and occupational accidents. Further research will be required to address this issue, especially for non-driving accidents at work.

There are several limitations in the current study. First, the response rate of the questionnaire survey was relatively low, which might have caused sample selection bias. However, the fact that the prevalence of high risk for OSA from this study was comparable to that from previous population-based studies suggests the validity of the sampling method of our study. Furthermore, because sleep habits were investigated based on self-report, quantitative data including sleep duration and sleep-wake cycles might be less accurate than measured by objective testing. However, perceived insufficient sleep has its own clinical significance in health outcomes separately from short sleep duration [24, 70], which supports the importance of subjective sleep evaluation. Finally, although we evaluated the associations of high risk for OSA with various factors, their causal relationship cannot be determined from this cross-sectional study.

## Supporting information

S1 File Raw data on all subjects. (XLSX)
